# Ultralong-Range Periodic Alignment and Structural Coloration of Titanate and Titanoniobate Nanosheets in Aqueous Dispersions

**DOI:** 10.1021/jacsau.5c01152

**Published:** 2025-10-10

**Authors:** Takayuki Kikuchi, Yasuo Ebina, Nobuyuki Sakai, Yoshiyuki Sugahara, Takayoshi Sasaki, Renzhi Ma

**Affiliations:** † Research Center for Materials Nanoarchitectonics (MANA), 52747 National Institute for Materials Science (NIMS), 1-1 Namiki, Tsukuba, Ibaraki 305-0044, Japan; ‡ Graduate School of Advanced Science and Engineering, Waseda University, 3-4-1 Okubo, Shinjuku-ku, Tokyo 169-8555, Japan; § Kagami Memorial Research Institute for Materials Science and Technology, Waseda University, 2-8-26 Nishi-waseda, Shinjuku-ku, Tokyo 169-0051, Japan

**Keywords:** structural coloration, liquid crystallinity, metal oxide nanosheets, surface charge, ionic strength

## Abstract

Structural coloration has been reported for many types of two-dimensional (2D) materials, including metal oxide nanosheets. In particular, metal oxide nanosheets feature a wealth of composition and structure, but their impact on structural color has not been explored. In the current study, we report the structural coloration observed for aqueous suspensions of three types of transition metal oxide nanosheets (Ti_1.73_O_4_^1.08–^, TiNbO_5_^–^, and Ti_2_NbO_7_^–^) obtained via the exfoliation of precursory layered compounds with tetramethylammonium hydroxide (TMAOH). It was revealed that a minute amount of TMA^+^ was retained even after deionization via repeated centrifugation, acting as countercations to the negatively charged nanosheets. These residual TMA^+^ ions contributed to stabilizing the dispersion of nanosheets under low-ionic-strength conditions, while the intersheet spacing was expanded to a magnitude comparable to the wavelength of the visible-light range, resulting in structural coloration. The wavelength of structural colors was dependent not only on nanosheet concentration but also on the type of nanosheets, correlating with the charge (ζ-potential) and thickness of the nanosheets, as well as the amount of TMA^+^ dissociated from the nanosheet surfaces. Specifically, TiNbO_5_^–^ was featured with the highest amount of TMA^+^ in the solution, which screened the negative charge of the nanosheets and resulted in the smallest intersheet spacing. On the other hand, when the dissociated amount of TMA^+^ was diminished, Ti_2_NbO_7_^–^, with a larger thickness, yielded a larger intersheet spacing than that of Ti_1.73_O_4_^1.08–^. These findings provide a valuable clue to the rational control of structural colors derived from nanosheets with different compositions and structures for a wide range of applications.

## Graphical abstract

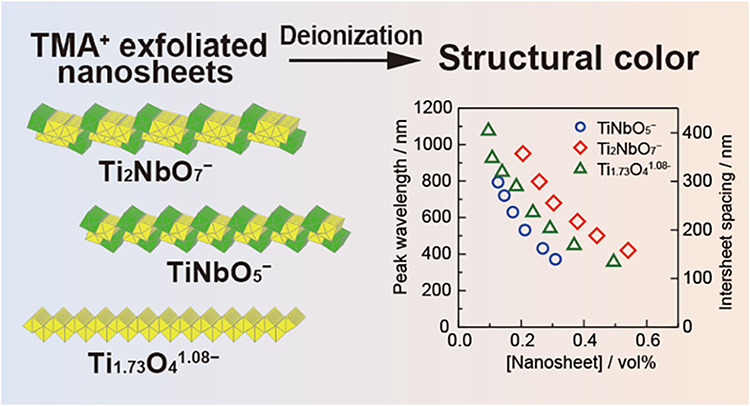

## Introduction

In 1949, Onsager proposed that materials with anisotropic shapes orderly arrange themselves in a solution to form liquid crystals to minimize the entropy in the system.^1^ For instance, if colloidal particles possess a large two-dimensional (2D) anisotropy, they tend to align cofacially and form nematic or lamellar liquid crystal phases. A study on the dispersion behavior of anisotropic particles, including 2D materials, is of great interest, as it could lead to rational control and improvement of the mechanical, thermal, optical, and electrical properties of colloidal/liquid crystalline materials.

On the other hand, it has been established that soft chemical processes could be applied to produce 2D materials with high aspect ratios in colloidal suspensions, so-called nanosheets. In 1998, our group reported the synthesis of titanate (Ti_0.91_O_2_^0.36–^) nanosheets with a thickness of 1 nm.^2,3^ In this soft chemical procedure, gigantic osmotic swelling of layered titanates is induced by the intercalation of a bulky organic amine, and subsequent exfoliation into monolayer nanosheets is attained by applying an external force. Such a synthetic method has been applied to various compounds to obtain a range of nanosheets with various compositions,^4,5^ including titanoniobate nanosheets.^6–8^ The dispersion behavior of the 2D nanosheets with anisotropic shapes was then studied. Nakato and Miyamoto reported that several kinds of oxide nanosheet suspensions exhibited liquid crystalline behavior.^9–11^ Typical periodic intersheet spacing of such liquid crystals was several tens of nanometers. Recently, Sano et al. reported the observation of structural color from titanate nanosheet suspensions with reduced ionic strength.^12^ Titanate nanosheets were found to align regularly at an ultralong intersheet distance of up to 675 nm, and the suspensions were able to reflect light from the UV to near-infrared (IR) range, depending on the nanosheet concentration. Surprisingly, the liquid crystal forms a centimeter-scale monodomain upon application of a strong magnetic field, exhibiting uniform and vivid structural color. Currently, liquid crystalline property and structural coloration have been confirmed for various 2D materials, e.g., graphene oxide,^13,14^ phosphate,^15–20^ hexaniobate,^21^ fluorinated clay,^22,23^ and layered perovskites.^24^ However, any other nanosheet dispersion that can reflect up to near-infrared light comparable to that of titanate nanosheets has not been reported except for a fluorinated clay.^22^ Such an unusual ultralong-range periodic alignment might be deeply related to the structural feature and resultant dispersion behavior of the nanosheets, but a comparative study of different types of nanosheets is not available.

In this study, three types of transition metal oxide nanosheets, titanate (Ti_1.73_O_4_^1.08–^) and titanoniobate (TiNbO_5_^–^ and Ti_2_NbO_7_^–^) nanosheets, were explored in terms of their structural colors. These nanosheets were selected as ideal model systems for comparative studies of structural color based on several important criteria, including high refractive index, transparency in the visible-light region, strong negative surface charge that enables stable dispersion, highly tunable composition, and ease of synthesis (synthetic accessibility). The nanosheets were derived from K_0.8_Ti_1.73_Li_0.27_O_4_, KTiNbO_5_, and CsTi_2_NbO_7_ via acid exchange and subsequent exfoliation with the tetramethylammonium hydroxide (TMAOH) solution, a common delaminating agent. The structural color was then observed after repeated sedimentation of nanosheets by centrifugation and redispersion with pure water. Structural colorations were examined by measuring the ion conductivity, pH, and ζ-potential values, as well as nuclear magnetic resonance (NMR) and UV–vis reflectance spectroscopy. Upon repeated deionization, a minute amount of dissociated TMA^+^ was retained, which acts as countercations of the nanosheets, allowing them to maintain their dispersed states. Furthermore, the Debye screening length increased with the removal of ions in the solution, resulting in an expansion of the intersheet spacing to a degree comparable to that in the visible-light wavelength range. This highly expanded lamellar structure induces the interference of visible light, leading to the evolution of structural colors. The wavelength of the structural color was influenced not only by the concentration of the nanosheets but also by their composition, which is correlated to their charge (ζ-potential) and thickness. At similar concentrations, the intersheet spacing increased in the order of TiNbO_5_^–^ (180 nm, 0.31 vol %), Ti_1.73_O_4_^1.08–^ (203 nm, 0.29 vol %), and Ti_2_NbO_7_^–^ (256 nm, 0.30 vol %). These findings thus offer valuable insights into the rational control of structural colors derived from 2D nanosheets with various compositions and structures, holding great potential for applications in reflective displays^25^ and chemical sensors.^12,26–29^

## Results and Discussion

Figure 1 shows atomic force microscopy (AFM) images and structure models of three types of metal oxide nanosheets, TiNbO_5_^–^, Ti_2_NbO_7_^–^, and Ti_1.73_O_4_^1.08–^. All nanosheets exhibit uniform molecular thicknesses of 1.5 nm for TiNbO_5_^–^ and Ti_2_NbO_7_^–^ and 1.2 nm for Ti_1.73_O_4_^1.08–^. The measured thickness can be understood in terms of the sum of the crystallographic thickness of the host layer (TiNbO_5_^–^: 1.05 nm, Ti_2_NbO_7_^–^: 1.06 nm, Ti_1.73_O_4_^1.08–^: 0.75 nm) and the size of surface-adsorbed species such as water molecules and/or TMA^+^ ions. This feature has been observed for a range of nanosheets and taken as evidence for the formation of single-layer nanosheets.^3,30^ AFM measurements were also performed after the adsorbed molecules were removed by UV irradiation and vacuum heating. As depicted in Figure S1 (Supporting Information), the thicknesses were reduced to 1.1 nm for TiNbO_5_^–^ and Ti_2_NbO_7_^–^ and 0.8 nm for Ti_1.73_O_4_^1.08–^. The values measured under high vacuum are in good agreement with the respective crystallographic thicknesses, reinforcing the interpretation that the increased thickness under ambient conditions originates from the presence of such surface species. AFM images over a wide area were collected and used to analyze the lateral size distribution of the nanosheets (Figure S2). The obtained histograms show that the average size of the nanosheets falls in a range of several hundred nanometers to a few micrometers, which is thousands of times larger than their thickness, yielding a high aspect ratio of approximately 1000. It is noteworthy that the as-exfoliated Ti_1.73_O_4_^1.08–^ nanosheets exhibit a much larger size (5–10 μm) than the other two types of nanosheets, which can be reduced by ultrasonic fragmentation (Figure S3). The sample after ultrasonic fragmentation for 2 h displays a size distribution comparable to those of TiNbO_5_^–^ and Ti_2_NbO_7_^–^ (Figure S2). To minimize the possible size effect on the structural coloration behavior, the fragmented Ti_1.73_O_4_^1.08–^ nanosheets were used in the following procedures.

**Figure 1:**
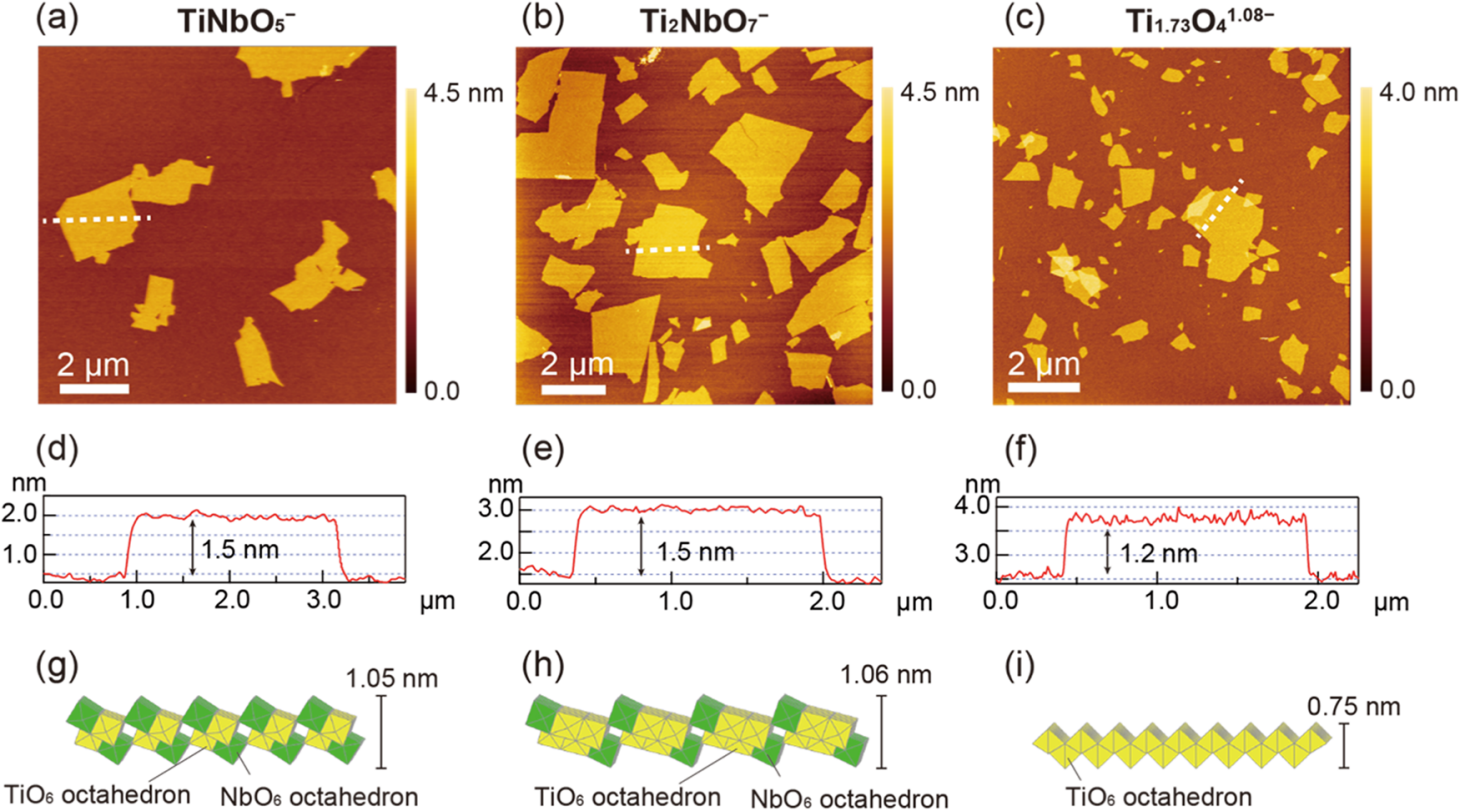
Typical AFM images of three types of nanosheets measured under ambient conditions: (a) TiNbO_5_^–^, (b) Ti_2_NbO_7_^–^, and (c) Ti_1.73_O_4_^1.08–^. The corresponding height profiles (d–f) and structures of the nanosheets (g–i).

TMA^+^ ions in the suspension were removed by settling out the nanosheets upon centrifugation and redispersion with water. To remove the electrolyte as much as possible, this process was repeated more than 10 times. The concentration of the nanosheets was readily adjusted in the final step by controlling the amount of pure water added for redispersion. The deionized suspension was injected into a homemade thin cell composed of quartz glass and a spacer to observe the structural color (Figure S4). The lamellar domains tended to align parallel to the cell face, as the nanosheets exhibited a cofacial orientation under the shear force generated by the injection into the cell. As shown in Figure 2, a vivid structural color appeared. It is obvious that these structural colors are derived from the Bragg reflection of the nanosheets separated by a long distance comparable to the visible-light wavelength range. The change in structural color and the corresponding shift in the peak of reflectance spectra were dependent on the concentration of nanosheets. In general, a deep blue color was observed at a high concentration of nanosheets. As the concentration decreased, the color became weaker due to a deteriorated orientation order of nanosheets, and the wavelength also red-shifted. In a dilute suspension, in which the intersheet spacing is sufficiently wide, multiple reflection peaks caused by higher-order Bragg reflections, such as second- and third-order reflections, may be observed in the visible region. For example, in the Ti_2_NbO_7_^–^ dispersion with a volume fraction of 0.21 vol %, the first-order peak was located in the infrared region at the wavelength of 951 nm, while the second-order peak fell in the visible region at 476 nm, yielding a light blue color. As dilution progressed further, the orientation order of the nanosheets gradually deteriorated, and the intensity of the reflection peak became weaker, eventually causing the disappearance of the structural color.

**Figure 2:**
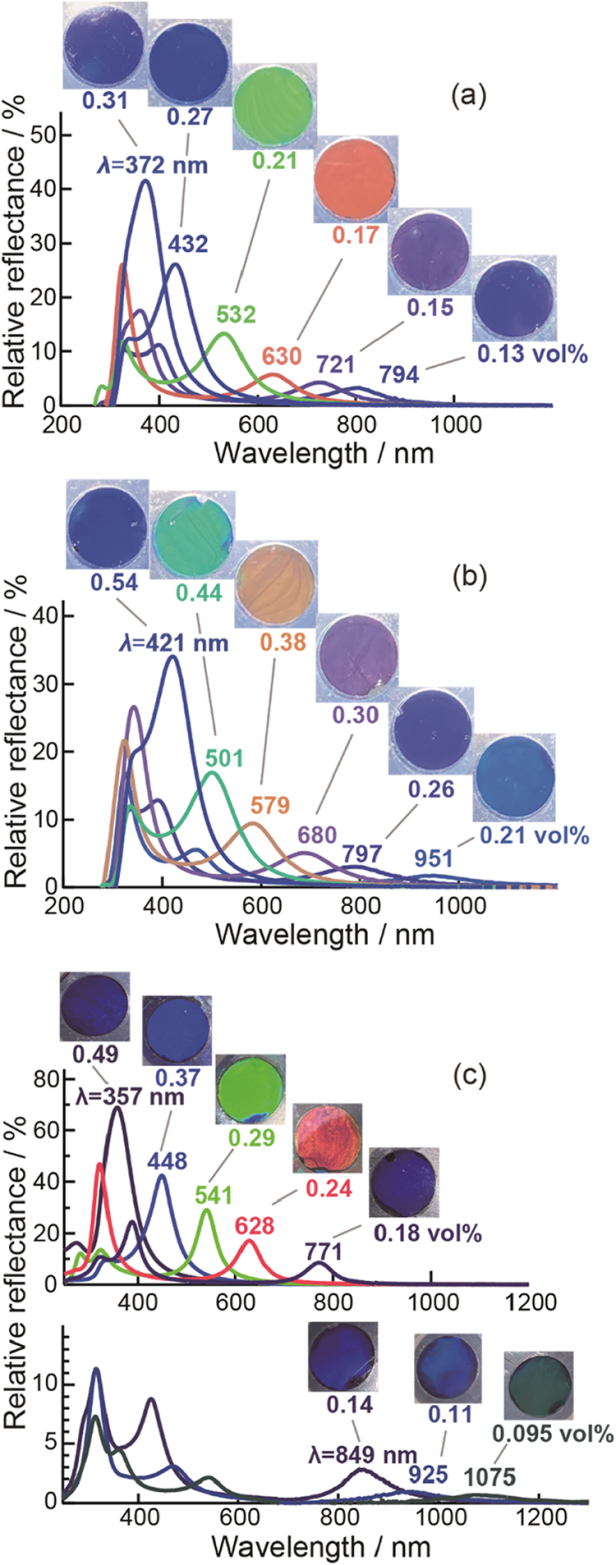
Appearance of structural colors observed from nanosheet suspensions and the corresponding reflectance spectra with different volume fractions (vol %) of nanosheets: (a) TiNbO_5_^–^, (b) Ti_2_NbO_7_^–^, and (c) Ti_1.73_O_4_^1.08–^. The peak wavelengths are labeled.

Figure 3 shows the relationship between the structural color and the nanosheet volume fraction in deionized suspensions. As schematically illustrated in Figure 3a, decreasing the nanosheet concentration expands the intersheet spacing, resulting in a red shift of the first-order Bragg reflection. The intersheet spacing (*d*) is calculated from the peak-top wavelength (λ) using Bragg’s equation (*m*λ = 2*n*_av_ *d* sin θ, where *m* represents diffraction order and *n*_av_ represents the averaged refractive index (1.33) of the suspension).^12^ As shown in Figure 3b, it is obvious that the intersheet spacings of the desalinated nanosheet suspensions vary for each type of nanosheet. For example, titanoniobate nanosheets with different compositions, TiNbO_5_^–^ and Ti_2_NbO_7_^–^, exhibit significantly different interlayer spacings even at the same volume fraction. Specifically, the periodic spacing of TiNbO_5_^–^ and Ti_2_NbO_7_^–^ suspensions containing the same total volume of nanosheets (0.21 vol %) deviates significantly over 150 nm. As TiNbO_5_^–^ yields the smallest intersheet spacing, the concentration range in which structural colors can be observed is also the narrowest.

**Figure 3:**
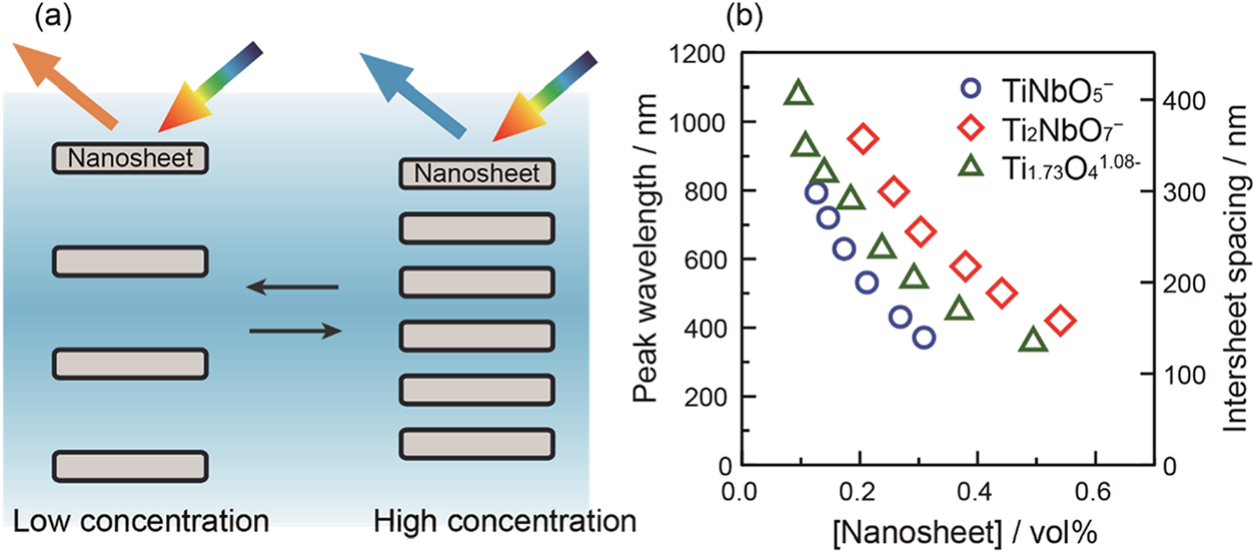
(a) Schematic illustration of the concentration-dependent change in intersheet spacing and the corresponding structural colors from the nanosheet suspensions. (b) First-order peak wavelengths of structural colors and the corresponding intersheet spacings observed from various deionized nanosheet dispersions. As the nanosheet volume fraction decreases, the intersheet spacing increases, resulting in a red shift of the Bragg reflection.

According to Onsager’s theory,^1^ when 2D materials form liquid crystals, the degree of orientation varies with their concentration and aspect ratio. It has been experimentally confirmed that at a high concentration, nanosheets tend to align uniaxially in the suspension. As the concentration decreases, the nanosheets gradually lose the orientation order.^31^ It is assumed that these nanosheets are fully oriented and packed into an ideal lamellar structure, as schematically illustrated in Figure 4a. The intersheet spacing can be estimated using the following equation.

1 $$d=t/\phi_{\mathrm{NS}}$$

where *t* is the thickness of the nanosheets and ϕ_NS_ is the volume fraction of the nanosheets.

**Figure 4:**
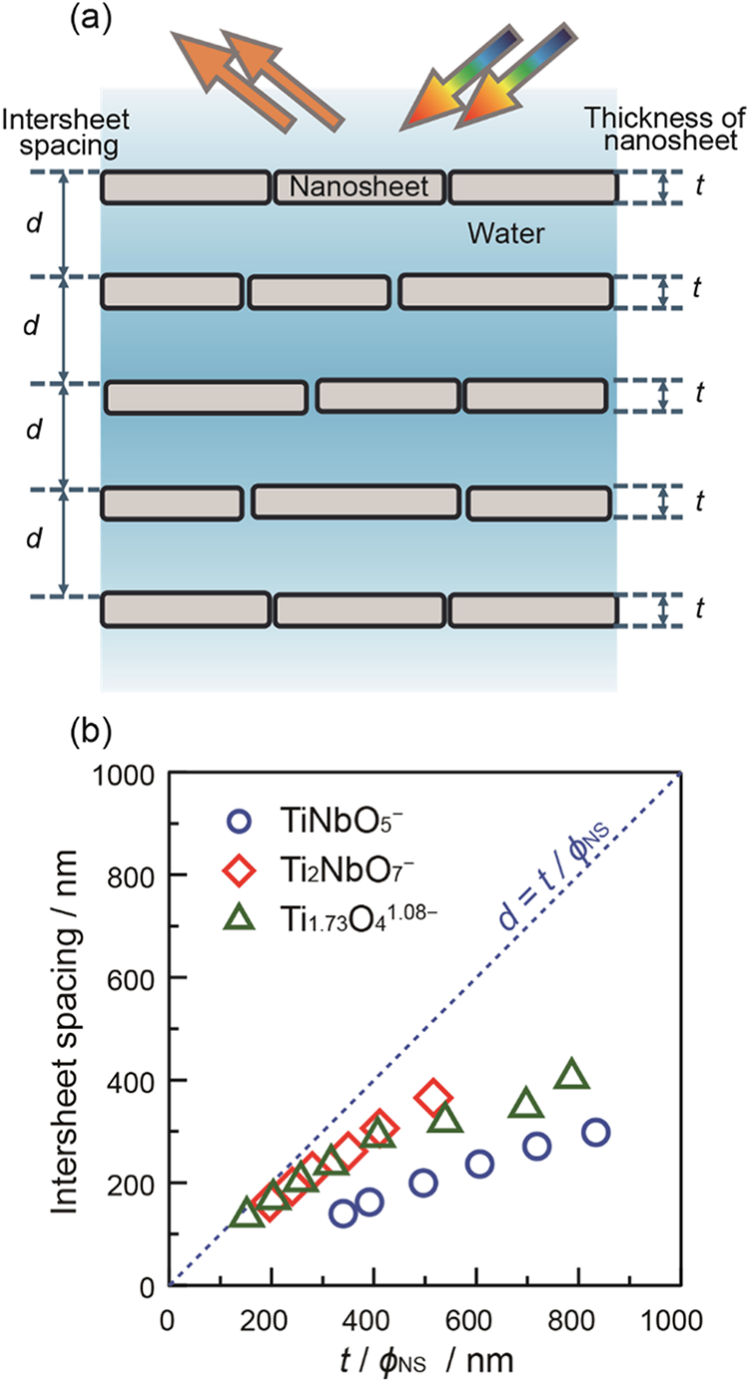
(a) Ideal lamellar structure model composed of aligned and packed nanosheets in a deionized suspension. The volume fraction of nanosheets (ϕ_NS_) can be estimated by intersheet spacing (*d*) and nanosheet thickness (*t*), ϕ_NS_ = *t*/*d*. (b) Dependence of measured intersheet spacing on the nanosheet thickness divided by the volume fraction. The dotted line represents a calculated intersheet spacing in the ideal lamellar model, *d* = *t*/ϕ_NS_.

Equation 1 indicates that the intersheet spacing is proportional to the inverse of the volume fraction. At the same nanosheet volume fraction, the intersheet spacing is proportional to the nanosheet thickness. As shown in Figure 4b, all measured intersheet spacings deviate downward from a dotted line representing the ideal lamellar model, as defined by eq 1. The deviation might be caused by the orientation disorder of the nanosheets or the insufficient development of lamellar domains. On the other hand, Sano et al. reported intersheet spacings in good agreement with eq 1 when Ti_1.73_O_4_^1.08–^ nanosheets with a large average size were aligned by a strong magnetic field.^12^ It implies that both size distribution and orientation enhancement of nanosheets may affect the intersheet spacing, which awaits further study. Integration of additional structural characterization methods, such as polarized optical microscopy (POM) and 2D-small angle X-ray scattering (SAXS) could provide valuable complementary insights into nanosheet alignment and lamellar ordering, which is currently being explored. As aforementioned, to minimize the size effect, all three types of nanosheets with comparable size distribution were employed in the current study. The plots of Ti_2_NbO_7_^–^ and Ti_1.73_O_4_^1.08–^ almost overlap with each other at the intersheet spacing between 160 and 300 nm. This suggests that Ti_2_NbO_7_^–^ and Ti_1.73_O_4_^1.08–^ nanosheets behave almost identically despite their different thicknesses (Ti_1.73_O_4_^1.08–^: 0.75 nm, Ti_2_NbO_7_^–^: 1.06 nm). On the other hand, although TiNbO_5_^–^ and Ti_2_NbO_7_^–^ nanosheets possess the same thickness, their plots deviate significantly. The smaller intersheet spacing of TiNbO_5_^–^ may be due to insufficient electrostatic repulsion. To explore this difference, the ionic strength of the nanosheet suspensions was further evaluated and compared.

Figure 5 shows the changes in pH and ionic conductivity during the deionization process. The volume fraction of nanosheets was unified at 0.04 vol %. In Figure 5a, the obtained pH values of the as-prepared nanosheet suspensions indicated strong basicity, which gradually decreased toward neutral as the hydroxide ions in the system were removed by repeating deionization. At the same time, some TMA^+^ ions also tend to detach from the nanosheet surface as the ionic strength decreases. However, a strong electrostatic attraction between TMA^+^ ions and the negatively charged nanosheets helps to retain a minimal amount of adsorbed TMA^+^ ions, even after repeated deionization. The residual TMA^+^ ions are essential in maintaining colloidal stability and suppressing the aggregation of nanosheets. After 10 cycles of deionization, all of the suspensions changed to weak base, with the pH value 9.0 for TiNbO_5_^–^, 8.0 for Ti_2_NbO_7_^–^, and 8.5 for Ti_1.73_O_4_^1.08–^. Figure 5b shows the change in ionic conductivity. The nanosheet suspension contains mainly TMA^+^ ions, which are the countercations to the negatively charged nanosheets, and residual hydroxide ions, as can be seen from the pH measurements. The ionic conductivity can thus be estimated from the mobility and number density of the nanosheets and the dissociated ions (TMA^+^, OH^–^) by the following equation:^32^

2 $$\sigma =n_{p}Z_{\mathrm{eff}}e\left(\mu_{p}+\mu_{H}\right)+\sum_{i}n_{i}e\mu_{i}$$

where σ is the ionic conductivity of the suspension; *n*_p_ and *n*_*i*_ are the number density of nanosheets and ions, respectively; *Z*_eff_ is the effective charge of nanosheets; *e* is the elementary charge; and μ_p_, μ_H_, and μ_*i*_ represent the mobility of nanosheets, adsorbed counterions, and dissociated ions, respectively.

**Figure 5:**
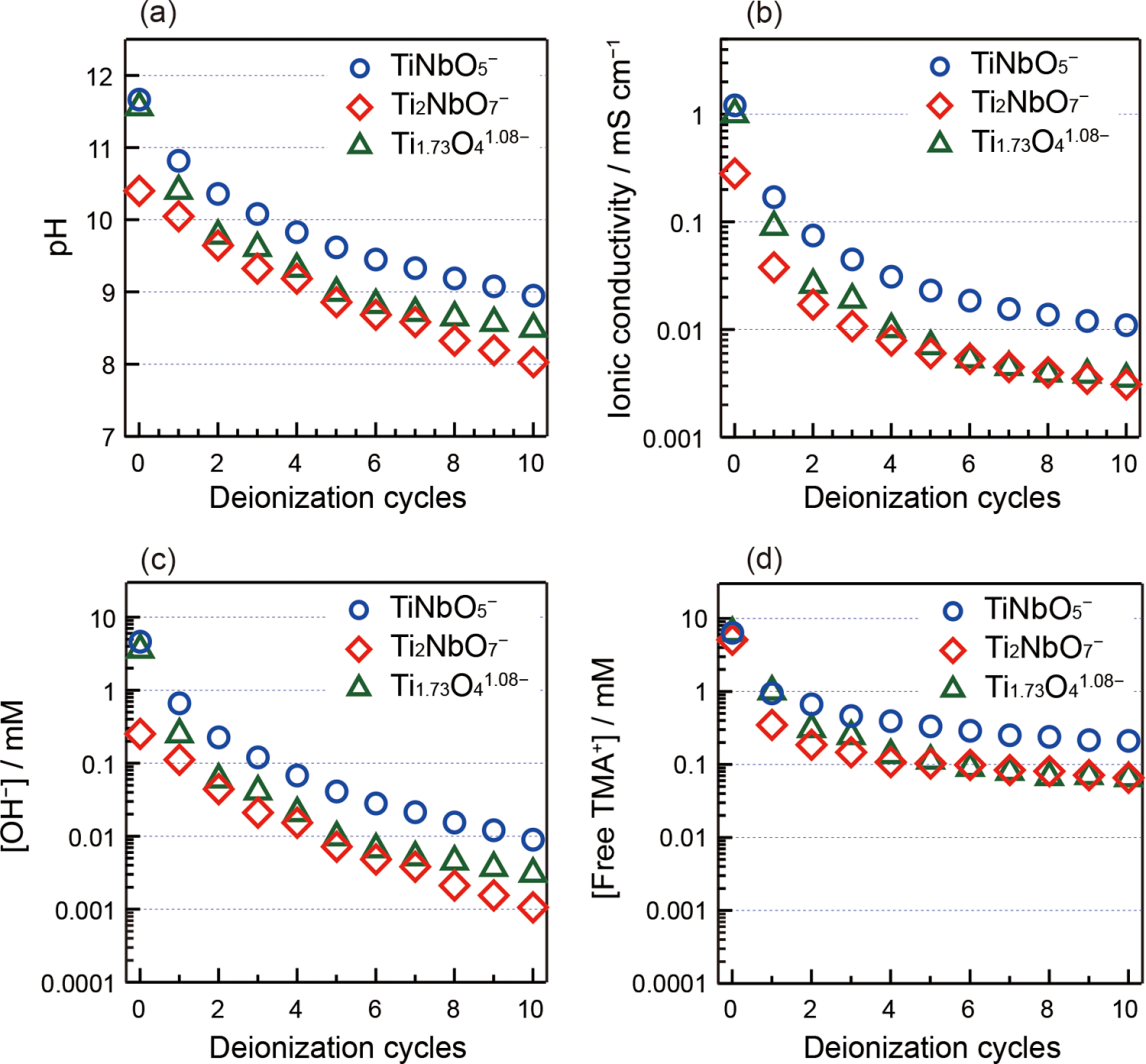
Changes in (a) pH and (b) ionic conductivity as functions of the number of deionization cycles. Suspensions of the same volume fraction of nanosheets, 0.04 vol %, were used. Each data point represents an averaged value of 4 samples. Variations in (c) hydroxide ion (OH^–^) and (d) TMA^+^ concentrations were estimated from the pH and ionic conductivity measurements.

The mobility values of TMA^+^ and OH^–^ are reported to be 4.66 × 10^–4^ and 20.64 × 10^–4^ cm^2^ V^–1^ s^–1^, respectively.^33^ On the other hand, the mobility of the nanosheets obtained from the ζ-potential measurements was in the range of 3.8 × 10^–4^ to 6.2 × 10^–4^ cm^2^ V^–1^ s^–1^, comparable to those of the ions. As the number density of Ti_1.73_O_4_^1.08–^ nanosheets with an average lateral size of 1 μm at 0.04 vol % is estimated to be 6.8 × 10^17^ m^–3^, which is sufficiently small compared to 6.0 × 10^20^ m^–3^ of dissociated ions at 1 μM, the contribution of the nanosheets to ion conductivity is negligible. Number density of dissociated TMA^+^ ions (*n*_TMA^+^_) can therefore be calculated as follows.

3 $$n_{{\mathrm{TMA}}^{+}}=\frac{\sigma -e\mu_{{\mathrm{OH}}^{-}}n_{{\mathrm{OH}}^{-}}}{e\mu_{{\mathrm{TMA}}^{+}}}$$

In eq 3, *n*_OH^–^_ can be obtained from the pH value. The variation of the OH^–^ and TMA^+^ concentrations is plotted in Figure 5c,d. It can be seen that, after repeated deionization, the concentrations of OH^–^ and TMA^+^ in the deionized TiNbO_5_^–^ suspension are higher than those of Ti_1.73_O_4_^1.08–^ and Ti_2_NbO_7_^–^.

The ionic strength (*I*) of the deionized nanosheet suspension can be determined from the concentrations of the dissociated TMA^+^ and OH^–^ by following eq 4:

4 $$I=\frac{1}{2}\sum C_{i}Z_{i}^{2}=\frac{1}{2}\left(\left[{\mathrm{TMA}}^{+}\right]+\left[{\mathrm{OH}}^{-}\right]\right)$$

where *C* represents the concentration of each ion and *Z* denotes the valence.

The calculated values of the three types of nanosheet suspensions are summarized in Table 1. It clearly shows that the ionic strength of TiNbO_5_^–^, 0.11 mM, is significantly larger than that of Ti_2_NbO_7_^–^ (0.033 mM) and Ti_1.73_O_4_^1.08–^ (0.034 mM).

**Table 1:** Ionic Environments of Deionized Nanosheet Dispersions with a Volume Fraction of 0.04 vol %^a^

	TiNbO_5_^–^	Ti_2_NbO_7_^–^	Ti_1.73_O_4_^1.08–^
nanosheet (NS) concentration (mM)	5.7	3.5	7.4
dissociated TMA^+^ (mM)	0.21	0.065	0.064
[OH^–^] (mM)	0.0090	0.0011	0.0031
ionic strength (mM)	0.11	0.033	0.034
ζ-potential (mV)	–46.2	–72.2	–74.5
adsorbed TMA^+^ [TMA_ad_^+^] (mM)	2.8	1.1	1.7
[TMA_ad_^+^]/[NS]	0.49	0.31	0.23
replacement rate of exchangeable protons	49%	32%	21%
percentage of exchangeable protons in protonic oxides	44.1%	37.5%	24.5%
charge density of nanosheets (*e* nm^–2^)	8.2	5.7	9.5

a [TMA_ad_^+^]/ [NS] represents the molar ratio of adsorbed TMA^+^ to nanosheets.

According to the Derjaguin–Landau–Verwey–Overbeek (DLVO) theory,^12,34^ a diffuse electric double layer may form in the vicinity of the charged nanosheets. When the nanosheets approach each other, the electric double layers start to overlap, causing repulsive forces and preventing aggregation. The thickness of this diffuse electric double layer is known as the inverse of the Debye screening length κ, which is proportional to the square root of ionic strength according to the following equation:

5 $$\kappa \propto \sqrt{\frac{I}{\varepsilon T}}$$

where ε is the dielectric constant of the suspension and *T* is the temperature.

In an ideal lamellar model, the intersheet spacing may be roughly estimated by the Debye screening length according to eq 6.^35^

6 $$d≅\frac{4}{\kappa }$$

From eqs 5 and 6, a smaller ionic strength broadens the diffuse electric double layer, resulting in the formation of a larger intersheet spacing. This tendency is consistent with the experimental results that the suspensions of Ti_2_NbO_7_^–^ and Ti_1.73_O_4_^1.08–^ exhibit an intersheet spacing larger than that of TiNbO_5_^–^. In addition, ζ-potential is another parameter representing the magnitude of electrostatic repulsion or attraction between the nanosheets, which is important in understanding the electrochemical equilibrium of the nanosheet dispersions. Again, larger ζ-potential values of Ti_2_NbO_7_^–^ and Ti_1.73_O_4_^1.08–^ nanosheets correspond well to the larger intersheet spacing than that of TiNbO_5_^–^.

As shown in Table 1, the total amount of TMA^+^ quantified by NMR is significantly larger than that of the dissociated TMA^+^ ions estimated by the ionic conductivity. The results indicate that approximately 1/5 to 1/2 of the exchangeable protons in HTiNbO_5_, HTi_2_NbO_7_, and H_1.08_Ti_1.73_O_4_ are replaced by TMA^+^ ions, which are mainly adsorbed on the negatively charged nanosheet surface. Due to strong electrostatic interactions between TMA^+^ ions and the adsorption sites, the adsorbed TMA^+^ are retained even after repeated deionization. Furthermore, the results are also consistent with the number and trend of exchangeable proton sites contained in the corresponding layered protonic oxides as evaluated by cation-exchange with ammonium acetate (Table S1). Therefore, the adsorption amount of TMA^+^ ions may reflect the inherent solid acidity of the nanosheets, which originates from the Brønsted acid sites associated with their metal–oxygen framework and local coordination structure.^7,36^ In the deionized suspensions, only a minute amount of TMA^+^ ions dissociates as free ions to form an electrical double layer. Such a low-ionic-strength condition enables the expansion of the intersheet spacing to a degree comparable to the visible wavelength range, resulting in structural coloration.

In the previous study on the swelling/delamination reaction of layered metal oxides with quaternary ammonium ions, a replacement rate of 34% of exchangeable protons in H_1.08_Ti_1.73_O_4_ was found at the equivalent dose of TMA^+^ ions,^37^ which is higher than the corresponding value of 21% as indicated in Table 1. To visualize the surface density of TMA^+^ ions adsorbed on the nanosheets, the area occupied by a single TMA^+^ ion is calculated based on each 2D rectangular lattice size and the replacement rate of exchangeable protons (see the Supporting Information). Figure 6 illustrates schematic models of adsorbed TMA^+^ ions on the host layer of the Ti_1.73_O_4_^1.08–^ swollen crystal in comparison with those on the surfaces of deionized nanosheets.^30,39^ For all of the deionized nanosheets, the occupied area exceeds the projected size of a TMA^+^ ion situated in a typical orientation,^37^ allowing gaps between them. As shown in Figure 6a,b, a comparison between the deionized nanosheet of Ti_1.73_O_4_^1.08–^ and the swollen crystal reveals that the density of TMA^+^ ions decreases following the deionization process. This result suggests that the repeated deionization process not only removes dissociated TMA^+^ ions in the suspension but also reduces the amount of adsorbed TMA^+^ ions, causing a decreased ratio to exchangeable protons.

**Figure 6:**
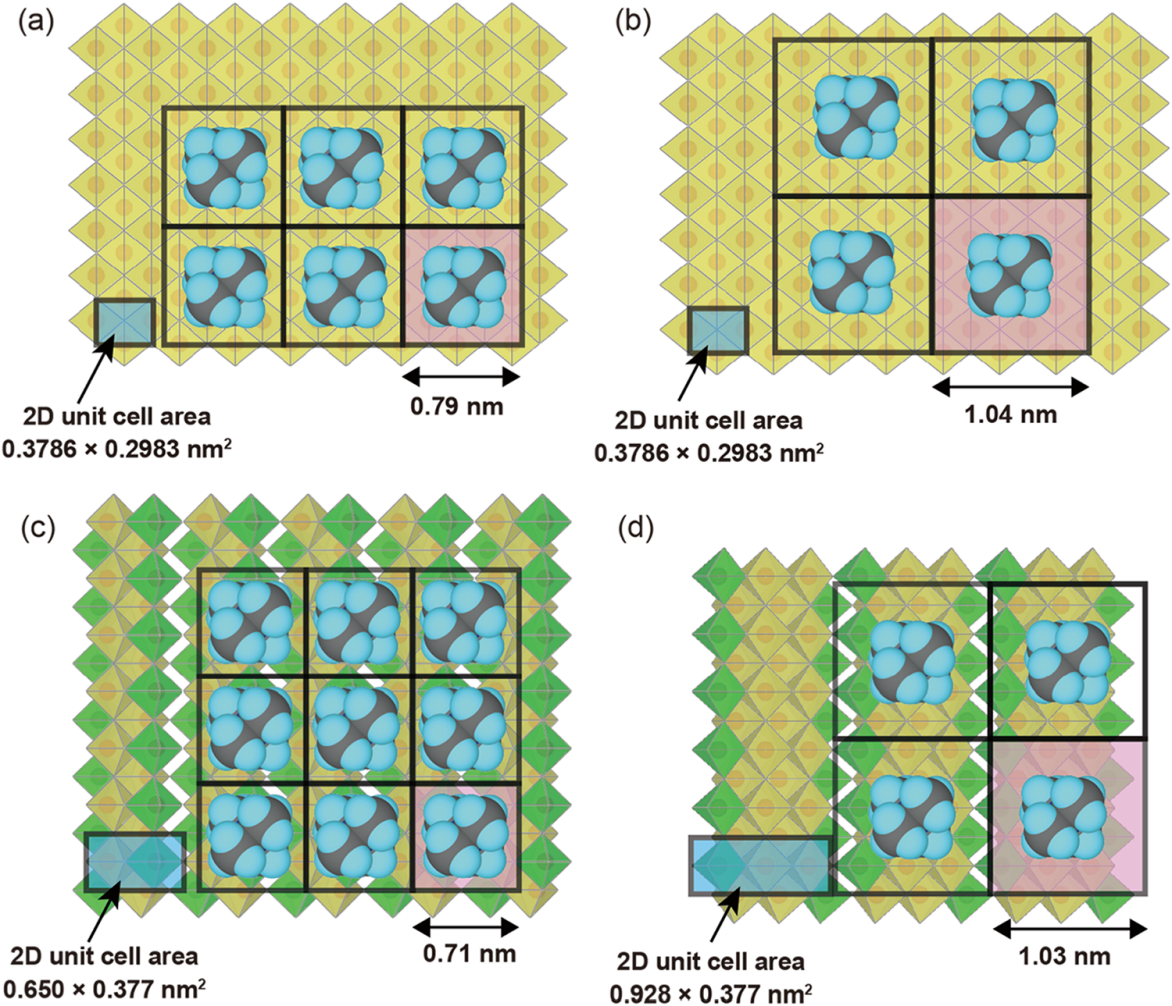
Schematic illustrations of adsorbed TMA^+^ ions on the host layer of (a) Ti_1.73_O_4_^1.08–^ swollen crystal^37^ and on the surfaces of (b) Ti_1.73_O_4_^1.08–^, (c) TiNbO_5_^–^, and (d) Ti_2_NbO_7_^–^ nanosheets after repeated deionization. The pink area in each panel indicates the projected area per one TMA^+^ ion based on the experimentally determined adsorption density. Panel a was adapted with permission from ref 37, Copyright 2024 Wiley-VCH. All crystal structures were visualized using VESTA.^38^

Moreover, the packing density of TMA^+^ ions on the surface of deionized Ti_2_NbO_7_^–^ is nearly equivalent to that on the deionized Ti_1.73_O_4_^1.08–^ nanosheet (Figure 6b,d), whereas the density on the surface of the deionized TiNbO_5_^–^ nanosheet apparently increases and appears comparable to that on the host layer of the Ti_1.73_O_4_^1.08–^ swollen crystal (Figure 6a,c). These results indicate that the area occupied by TMA^+^ under low-ionic-strength conditions varies, depending on the type of nanosheets. The variation in TMA^+^ adsorption can be related to its intrinsic structural charge characteristics. As indicated in Table 1, charge densities of the nanosheets were calculated based on the number of compensating cations required to neutralize the net negative charge per unit cell, which also reflects the available acidic sites. For Ti_2_NbO_7_^–^ and TiNbO_5_^–^, the measured TMA^+^ amount increases with the surface charge increasing from 5.7 to 8.2 *e* nm^–2^. Although steric hindrance may prevent the stoichiometric adsorption of TMA^+^ on every available site, the observed trend suggests a possible correlation between charge density and TMA^+^ uptake. On the other hand, Ti_1.73_O_4_^1.08–^ yields the lowest adsorption value despite the highest charge density (9.5 *e* nm^–2^). This discrepancy might be attributed to the peculiar lepidocrocite-type structure as shown in Figure S6, where Ti vacancies (2.37 sites/nm^2^) can act as preferential binding sites for cationic species.^40^ In addition, it has been reported that protonic titanoniobates generally exhibit stronger Brønsted acidity than titanates, due to the incorporation of a higher valent central metal (Nb^5+^).^100,101^ Therefore, the adsorption behavior appears to be dictated by a combination of factors, including the solid acidity, charge density, and number of adsorption sites, as well as the steric compatibility. From Table 1, it seems that the quantity of dissociated TMA^+^ generally correlates with the amount adsorbed on the nanosheet surface, resulting in the highest ionic strength of the deionized suspension of TiNbO_5_^–^.

The above analyses thus indicate that the intersheet spacing is mainly determined by ionic strength, i.e., the Debye screening length of the deionized suspension, which is directly related to the structure of the nanosheets. For example, the differences in charge and thickness could qualitatively explain why TiNbO_5_^–^ exhibits a relatively smaller intersheet spacing than those of Ti_2_NbO_7_^–^ and Ti_1.73_O_4_^1.08–^.

Previous results have demonstrated that structural colors in nanosheet suspensions responded sensitively to changes in the chemical environment, such as pH-responsive behavior reported for Ti_1.73_O_4_^1.08–^.^12^ As shown in Figure S7, the pH responses of TiNbO_5_^–^ and Ti_2_NbO_7_^–^ suspensions were evaluated. For the Ti_2_NbO_7_^–^ suspension, as pH approached neutrality, the reflection peak shifted toward a shorter wavelength (blue shift), and the reflection intensity gradually decreased. This suggests that the protonation of the nanosheet surface and the partial desorption of TMA^+^ accompanying the neutralization of the suspension induced a decrease in electrostatic repulsion between nanosheets, resulting in a reduction in the intersheet spacing and a decrease in the orientation order. On the other hand, a similar decrease in reflection intensity was observed for the TiNbO_5_^–^ suspension, but the change in the reflection peak wavelength was not very evident. This behavior implies that the electrostatic repulsion between nanosheets was largely maintained without any significant reduction in intersheet spacing. The decrease in reflection intensity may be due to increasing orientational disorder of the nanosheets, but the exact cause remains to be explored. Nevertheless, the above results indicate that the pH-responsive behavior is dependent on the type and surface characteristics of the nanosheets, which may be useful for a tailored design of optical sensors.

A comparison summarizing the structural coloration behavior of representative 2D materials is shown in Table S2. Among them, transition metal oxide nanosheets exhibit a notably wide reflection wavelength range and high reflectance, demonstrating their potential as high-performance structural color materials. In addition to the nontoxic nature, the high refractive index and low background absorption of transition metal oxide nanosheets are beneficial in the realization of high reflection intensity and clear color tones. The high surface charges also enable dispersion stability without aggregation across a wide concentration range, generating highly tunable intersheet spacing and the resultant structural colors. The quantitative evaluation and comparative study of multiple metal oxide nanosheets presented in this study provide valuable insights, as well as general guidelines for the rational control of structural colors based on various 2D materials with different compositions and structures.

## Conclusions

A comparative study on structural colors obtained from three types of transition metal oxide nanosheets was conducted. It was found that the nature of the structural colors is dependent on the structure of the nanosheets and the ionic strength of the resulting suspension. In general, larger ionic strength results in smaller intersheet spacing due to the effective shielding of electrostatic repulsive forces, leading to a blue shift of the structural color or even the disappearance of the visible structural color. Upon repeating the deionization process of the nanosheet suspensions, the ionic strength is diminished with the decreased concentrations of TMA^+^ and hydroxide ions. The concentration of dissociated ions as well as the amount of TMA^+^ adsorbed on the nanosheet surface are strongly correlated with the charge feature of the nanosheets. This could well explain the result that TiNbO_5_^–^ nanosheets exhibit the smallest intersheet spacing in comparison with Ti_2_NbO_7_^–^ and Ti_1.73_O_4_^1.08–^. Moreover, when sufficient electrostatic repulsion is attained at the same volume fraction of nanosheets with different thicknesses, a larger intersheet spacing and the corresponding longer wavelength of structural color were yielded for thicker nanosheets, such as Ti_2_NbO_7_^–^. While previous studies have focused on a single type of nanosheet, the current study covers multiple metal oxide nanosheets with different compositions to verify the mechanism of structural color formation. The comparative exploration of plausible models and the measured values (such as TMA^+^ adsorption, exchangeable proton quantity, and ζ-potential) greatly deepens the understanding of the macroscopic ordering and/or liquid crystallinity of nanosheet suspensions.

Structural coloration of colloidal nanosheets is not only promising in chromic technology highly sensitive to even small environmental changes, but it is also attractive in exploring multifunctions derived from 2D materials. Transition metal oxide nanosheets are known for versatile properties, such as high UV absorption, photocatalytic activity, and a superior dielectric constant. The important insights revealed in the current study may help accelerate the research on fabricating mechano-thermo-photochromic devices based on colloidal nanosheets.

## Experimental Section

### Preparation of Titanoniobates and Titanate Nanosheet Dispersions

The nanosheet suspensions were synthesized according to procedures reported previously.^3,41–43^ Layered metal oxide polycrystals of KTiNbO_5_, CsTi_2_NbO_7_, and K_0.8_Ti_1.73_Li_0.27_O_4_ were synthesized via a solid-state reaction. These polycrystals were proton-exchanged to form HTiNbO_5_, HTi_2_NbO_7_·2H_2_O, and H_1.08_Ti_1.73_O_4_·H_2_O by the treatment with HCl and then delaminated by vigorously shaking with an aqueous TMAOH solution (TMA^+^/H^+^ = 1.0). The resulting suspensions were subjected to centrifugation at an appropriate speed to remove the unexfoliated materials. The nanosheet suspension was diluted and dropped onto a clean silicon substrate. After drying, AFM observation was carried out to characterize the thickness and lateral size of the nanosheets. The volume fractions of the nanosheets were calculated from their molar concentration, as reported in the literature.^11^ The molar concentrations were determined by gravimetric analysis of the powder that remained after drying the nanosheet suspension, followed by heating at 1000 °C.

### Deionization Process and Structural Color Measurement

As previously reported,^12^ excessive ions in the suspension were removed by the following deionization procedure. The centrifuge tube containing 25 cm^3^ of nanosheet dispersion was centrifuged (15 000 rpm, 1 h), settling out all dispersed nanosheets in the suspension. The supernatant was removed using a pipette. The same volume of pure water was added, and the sediment was redispersed by stirring with a vortex mixer. This operation was typically repeated 15 times for titanate nanosheets and 10 times for the other two titanoniobates until the ionic conductivity was below 0.01 mS cm^–1^, which is comparable to that of a 0.1 mM TMAOH aqueous solution. The deionized suspension was injected into a homemade thin quartz glass cell with a gap of 0.13 mm (see Figure S4). The reflectance spectra of the suspensions sealed in the cells were measured with a UV–vis spectrophotometer. A standard white reflector was used as a baseline for the measurements, and a spectrum from the same cell filled with pure water was used to cancel the absorption by the solvent.

### Characterization of Deionization Process in Nanosheet Dispersion

A change in the concentration of each ion in the suspension during the deionization process was monitored by pH, ion conductivity, and ^1^H NMR measurements. The hydroxide ions in the suspension were determined by pH measurement. As the nanosheet suspension contains mainly TMA^+^ and hydroxide ions as free ions, the concentration of dissociated TMA^+^ was calculated by using the ion conductivity data as well as the amount of hydroxide ions obtained from the pH measurement. ^1^H NMR was used to quantify the concentration of TMA^+^ in the suspension by comparing the area of the peak of TMA^+^ and DMSO added as an internal standard. This value is the sum of dissociated and adsorbed TMA^+^, and the concentration of TMA^+^ adsorbed on the nanosheets was calculated by subtracting the concentration of free ions. The ζ-potential of the deionized nanosheet suspension was measured by electrophoretic light scattering, and the average value obtained from at least five measurements was used as the ζ-potential. The concentration was unified at 0.04 vol % by diluting the original nanosheet suspension with pure water. To evaluate the pH-responsive behavior, a diluted hydrochloric acid solution (20 μM for Ti_2_NbO_7_^–^ or 200 μM for TiNbO_5_^–^) was added dropwise to the deionized suspensions. The changes in the reflection spectrum were monitored.

## Supplementary Material



## Notes

Material characterizations, additional AFM images and lateral size histograms, structural color observation, estimation of the area occupied by TMA^+^ ions on the nanosheet surface, estimation of the exchangeable proton content in layered protonic oxides, cross-sectional schematic illustrations of the nanosheet structures, pH responses of structural colors, and comparison of the structural colors of 2D materials (PDF)
