# Supplementary material for: Ultralong-Range Periodic Alignment and Structural Coloration of Titanate and Titanoniobate Nanosheets in Aqueous Dispersions
Source: JACS Au. 2025 Oct 10;5(10):5207–16. doi: 10.1021/jacsau.5c01152 (PMC12569687; doi:10.1021/jacsau.5c01152)
Supplement: Supplementary file 1 [file au5c01152_si_001.pdf]

Supporting information

Ultralong-Range Periodic Alignment and Structural Coloration of Titanate and Titanoniobate Nanosheets in Aqueous Dispersions

Takayuki Kikuchi,[1,2] Yasuo Ebina,[1] Nobuyuki Sakai,[1] Yoshiyuki Sugahara,[2,3] Takayoshi Sasaki,[1*] and Renzhi Ma[1,2*]

[1] *Research Center for Materials Nanoarchitectonics (MANA), National Institute for Materials Science (NIMS), 1-1 Namiki, Tsukuba, Ibaraki 305-0044, Japan*

[2] *Graduate School of Advanced Science and Engineering, Waseda University, 3-4-1 Okubo, Shinjuku-ku, Tokyo 169-8555, Japan*

[3] *Kagami Memorial Research Institute for Materials Science and Technology, Waseda University, 2-8-26 Nishi-waseda, Shinjuku-ku, Tokyo 169-0051, Japan*

*Corresponding authors: ma.renzhi@nims.go.jp (R. Ma); sasaki.takayoshi@nims.go.jp (T. Sasaki).

Sample Characterization.

XRD data were collected using Rigaku ULTIMA IV powder diffractometer with a graphite monochromatized Cu Kα radiation (λ = 0.15405 nm). AFM images of nanosheets deposited on Si substrates were obtained using two instruments: Hitachi SPI3800N/SPA-400 under ambient conditions and Hitachi AFM5000II/AFM5300E/E-sweep under vacuum conditions (10^{-4} Pa). In both cases, measurements were conducted in noncontact mode using a Si cantilever (SI-DF20). For removal of adsorbed species from nanosheet surface via UV irradiation, San-Ei Electric UVE-502S/UVC502S was used. For centrifugation, an Eppendorf Himac CP80NX ultracentrifuge and a P70AT rotor were used. Ultrasonication was performed using Kaijo Sono Cleaner 200D. Reflection spectra were recorded using Shimadzu SolidSpec-3700 DUV. Ion conductivity measurements were performed using a LAQUA DS-71 ion conductometer (HORIBA). For ^{1}H-NMR measurements, equal amounts of DMSO solution diluted 2500 times with heavy water and a nanosheet suspension were mixed and measured with a JEOL ECZ-400. The molar concentration of TMA^+ is obtained by comparing the peak area of DMSO as an internal standard. For zeta potential measurements, an ELSZ-2 (Otsuka Electronics) equipped with a high-concentration cell unit was employed. The applied voltage was set to 5 V, and zeta potential was calculated based on the Smoluchowski equation. For pH measurement, PURE IL 9600-10D electrode and F-2000PI tabletop pH/water analyzer (HORIBA) were used.

AFM Characterization of Nanosheets in Vacuum.

AFM measurements were conducted on nanosheets after removal of surface-adsorbed species via UV irradiation and vacuum heating. Aqueous dispersions of $Ti_{1.73}O_4^{1.08-}$ nanosheets were drop-cast onto Si substrates, followed by exposure to UV light from a high-pressure mercury lamp at an intensity of 90 mW/cm^2 (< 340 nm) for 15 min. Subsequently, the substrates were heated at 300 °C for 1 h in a high vacuum chamber equipped with AFM. The samples were then cooled and AFM observation was performed at room temperature.

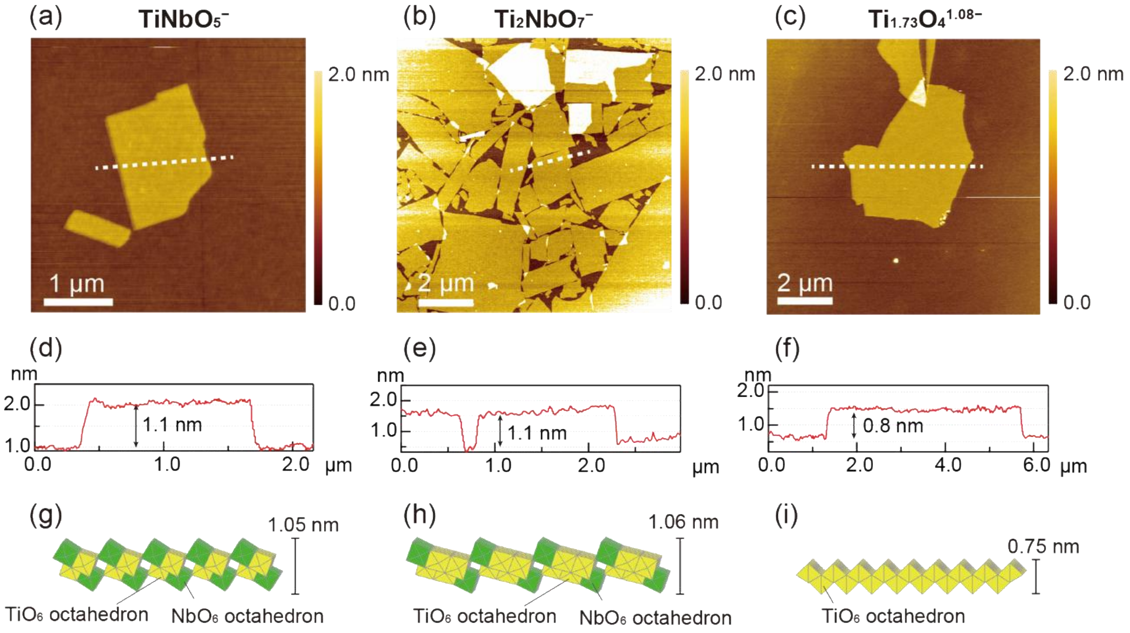

Figure S1. Typical AFM images of three types of nanosheets measured under vacuum conditions. (a) $TiNbO_5^-$; (b) $Ti_2NbO_7^-$; (c) $Ti_{1.73}O_4^{1.08-}$. Corresponding height profiles (d-f) and structures of the nanosheets (g-i).

Lateral Size Distributions of Nanosheets.

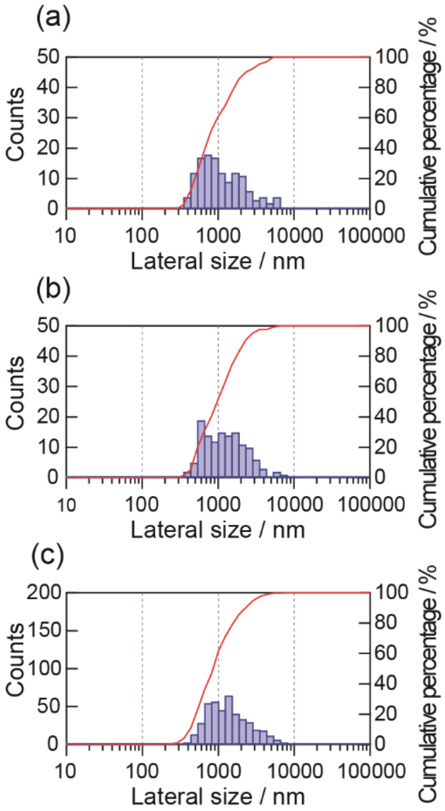

Figure S2. Histograms of lateral size: (a) $TiNbO_5^-$; (b) $Ti_2NbO_7^-$; (c) $Ti_{1.73}O_4^{1.08-}$ (sonicated 2 h). The lateral size was approximated as the diameter of a circle with an area equivalent to that of individual nanosheets. The red line indicates the cumulative percentage.

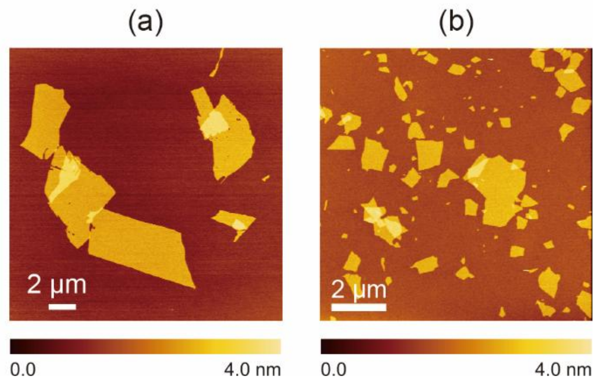

Figure S3. Representative AFM images of (a) as-exfoliated $Ti_{1.73}O_4^{1.08-}$ nanosheets and (b) after ultrasonic fragmentation for 2 h.

Structural Color Observation.

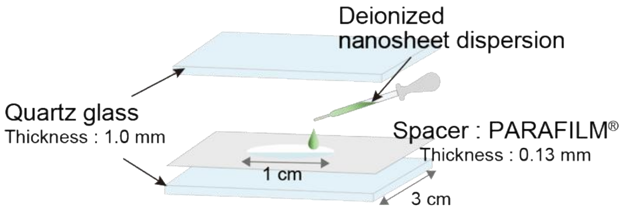

Figure S4. Schematic illustration of a home-made thin cell for structural color observation.

The cell thickness was experimentally optimized to ensure both optical clarity and stable shear-induced alignment of nanosheets. A thinner cell led to insufficient sample volume, making it difficult to observe vivid structural color or acquire reliable reflectance spectra. A thicker cell, on the other hand, resulted in non-uniform shear flow and poor nanosheet alignment.

Estimation of the Area Occupied by TMA$^+$ Ions on Nanosheet Surface.

The area occupied by a single TMA$^+$ ion on each nanosheet surface was estimated following the previous report.[1] Figure 6 illustrates the top view of the nanosheets, where the size of the 2D rectangular unit cell ($S_{\text{unit cell}}$) varies depending on the composition of each nanosheet. Each unit cell contains two structural units of [$Ti_{1.73}O_4^{1.08-}$], [$TiNbO_5^-$], or [$Ti_2NbO_7^-$], and bears either approximately 1.08 or 1 negative charge.

Assuming the theoretical ion-exchange capacity to be x, the area per TMA$^+$ ion (S) can be calculated as follows.

$S = 2 \times S_{\text{unit cell}} / x$

Here, the factor of 2 accounts for the fact that both the top and bottom surfaces of the nanosheets are available for ion adsorption. The theoretical ion-exchange capacity x was taken from the "Replacement rate of exchangeable protons" listed in Table 1.

Estimation of Exchangeable Proton Content in Layered Protonic Oxides.

The exchangeable proton content in protonated layered metal oxides was estimated by a cation-exchange reaction with ammonium acetate.[2] Approximately 0.1 g of powdered sample was placed in a 50 mL Erlenmeyer flask, and 25 mL of 0.1 M ammonium acetate aqueous solution was added. The mixture was stirred from time to time at 25 °C and left to stand for 24 h, after which the supernatant was collected and its pH was measured. pH titrations were also conducted by adding various amounts of acetic acid to 25 mL of 0.1 M ammonium acetate buffer solution to obtain a calibration curve. The amount of protons exchanged with NH_4^+ ions was calculated using the obtained calibration curve.

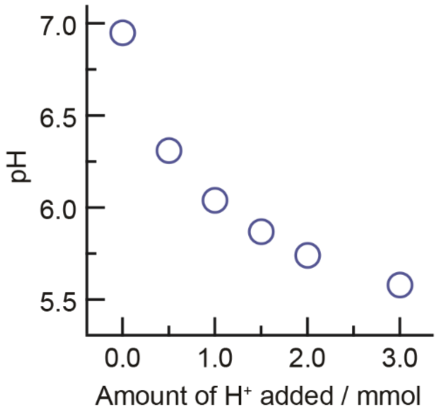

Figure S5. pH titration curve of ammonium acetate buffer solution.

Table S1. Quantification of exchangeable protons in protonated layered metal oxides.

	$HTiNbO_5$	$HTi_2NbO_7{\cdot}2H_2O$	$H_{1.08}Ti_{1.73}O_4{\cdot}H_2O$
Sample weight / g	0.101	0.110	0.101
Mole / µmol	451	326	603
pH of supernatant	5.75	5.95	5.90
Exchanged H^+ / µmol	199	122	138
Percentage of exchangeable protons	44.1%	37.5%	24.5%

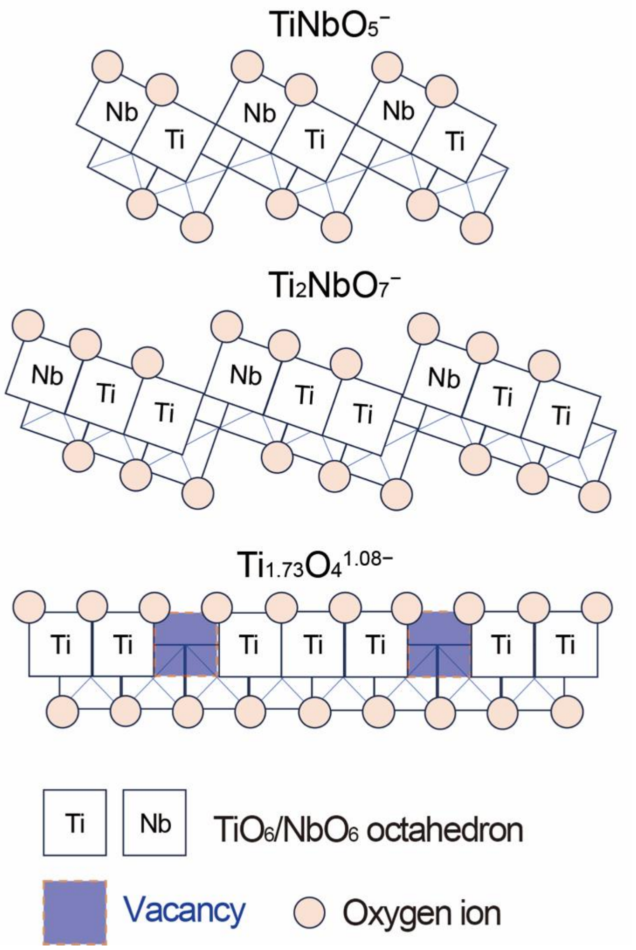

Figure S6. Cross-sectional schematic illustrations of the nanosheet structures.

pH-Responsive Behavior of Structural Colors.

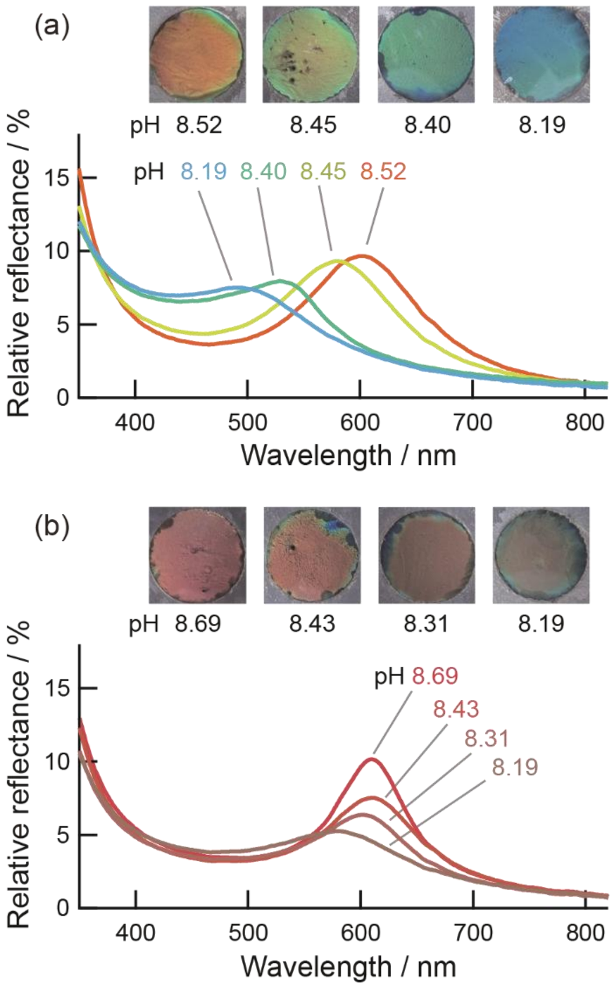

Figure S7. Appearance of structural colors observed from nanosheet suspensions and corresponding reflectance spectra with different pH values: (a) $Ti_2NbO_7^-$ (0.37 vol%); (b) $TiNbO_5^-$ (0.17 vol%).

Table S2. Comparison of structural colors of 2D materials.

Materials	Concentration range / vol%	Reflection wavelength range / nm	Reflectance / %	Alignment enhancement	Reference
Antimony phosphate	0.21-0.42	400-510	15% @ 400 nm		3
Cs-fluorohectorite	0.34-1.34	400-700	25% @450 nm		4
Fluorohectorite	(0.6-1.9 wt%)	327-900			5
Graphene-oxide	0.13-0.50	350-700	3% @540 nm		6
Graphene-oxide	0.27-0.69	338-632			7
$Ti_{1.73}O_4^{1.08-}$	0.09-0.50	370-1750		Magnetic field	8
$TiNbO_5^-$	0.13-0.31	372-794	27% @ 432 nm		This work
$Ti_2NbO_7^-$	0.21-0.54	421-951	35% @421 nm		This work
$Ti_{1.73}O_4^{1.08-}$	0.095-0.49	357-1075	40% @ 448 nm		This work

REFERENCE

(1) Song, Y.; Sakai, N.; Ebina, Y.; Iyi, N.; Kikuchi, T.; Ma, R.; Ishida, Y.; Sasaki, T. Systematic Study on Swelling/Delamination of Layered Metal Oxides with Quaternary Ammonium Ions: Production of Well-Shaped/Oversized Unilamellar Nanosheets. *Small Methods* **2025**, *9*, 2401055.

(2) Holm, V. C. F.; Bailey, G. C.; Clark, A. Acidity Studies of Silica-Alumina Catalysts. *J. Phys. Chem*. **1959**, *63*, 129.

(3) Mouri, E.; Fukumoto, T.; Kato, R.; Miyamoto, N.; Nakato, T. Time Evolution of the Inner Structure of Antimony Phosphate Nanosheet Suspension Developing Structural Colouration. *Soft Matter* **2024**, *10*, 6353.

(4) Michels-Brito, P. H.; Dudko, V.; Wagner, D.; Markus, P.; Papastavrou, G.; Michels, L.; Breu, J.; Fossum, J. O. Bright, Noniridescent Structural Coloration from Clay Mineral Nanosheet Suspensions. *Sci. Adv.* **2022**, *8*, eab18147.

(5) Miyamoto, N.; Yamamoto, S. Angular-Independent Structural Colors of Clay Dispersions. *ACS Omega* **2022**, *7*, 6070–6074.

(6) Shen, T.-Z.; Hong, S.-H.; Lee, B.; Song, J.-K. Bottom-Up and Top-Down Manipulations for Multi-Order Photonic Crystallinity in a Graphene-Oxide Colloid. *NPG Asia Mater.* **2016**, *8*, e296.

(7) Li, P.; Wong, M.; Zhang, X.; Yao, H.; Ishige, R.; Takahara, A.; Miyamoto, M.; Nishimura, R.; Sue, H.-J. Tunable Lyotropic Photonic Liquid Crystal Based on Graphene Oxide. *ACS Photonics* **2014**, *1*, 79–86.

(8) Sano, K.; Kim, Y. S.; Ishida, Y.; Ebina, Y.; Sasaki, T.; Hikima, T.; Aida, T. Photonic Water Dynamically Responsive to External Stimuli. *Nat. Commun.* **2016**, *7*, 12559.
